# Cognitive performance and stroke-specific quality of life four years after stroke

**DOI:** 10.3389/fresc.2025.1643004

**Published:** 2025-10-09

**Authors:** Marte C. Ørbo, Oddgeir Friborg, Audny Anke, Marianne Berg Halvorsen, Mari Thoresen Løkholm, Synne Garder Pedersen

**Affiliations:** ^1^ Department of Psychology, Faculty of Health Sciences, UiT—The Arctic University of Norway, Tromsø, Norway; ^2^ Department of Rehabilitation, University Hospital of North Norway, Tromsø, Norway; ^3^ Faculty of Medicine, Institute of Health and Society, Research Centre for Habilitation and Rehabilitation Models and Services (CHARM), University of Oslo, Oslo, Norway; ^4^ Department of Clinical Medicine, Faculty of Health Sciences, UiT—The Arctic University of Norway, Tromsø, Norway; ^5^ Vigor Rehabilitation Hospital, Tromsø, Norway

**Keywords:** stroke, cognition, verbal memory, health-related quality of life, reaction time, fine-motor coordination

## Abstract

Long-term cognitive outcomes after stroke and their impact on health-related quality of life remain understudied. This study examined associations between cognitive performance and the Stroke-Specific Quality of Life scale (SS-QOL) four years after stroke. Sixty-five individuals (mean age 64 years, 74% male) with mild-to-moderate strokes completed the SS-QOL, the Modified Rankin Scale (mRS) and a neuropsychological test battery. A previously established principal component analysis of the SS-QOL informed division into Cognitive-Social-Mental (CSM) and Physical-Health (PH) components. Most participants reported no or mild disability on the mRS. Relative to age-adjusted norms, the group performed slightly below average across several cognitive domains, with marked variability indicating a subgroup with pronounced deficits. PH scores were high, reflecting minimal physical disability, whereas CSM scores were lower, indicating persistent challenges. CSM scores were associated with reaction time (*ρ* = .47), verbal memory (*ρ* = .42) and fine-motor coordination (*ρ* = .39; all *p* ≤ *.001*). PH scores were associated with fine-motor coordination (*ρ* = .49*; p* *<* *.001*). No significant associations emerged for language, visuospatial abilities, attention or executive functions after correction for multiple comparisons. In summary, associations between cognitive domains and SS-QOL were circumscribed and concentrated within the CSM component. Results indicate that cognitive and psychosocial factors are relevant in long-term recovery. Even selective cognitive deficits could reduce health-related quality of life and warrant follow-up. Generalisability is limited by the small, predominantly male sample, exclusion of individuals with aphasia, severe disability or age >75 years. Replication in larger, more diverse samples is needed.

## Introduction

Stroke remains a leading cause of disability and mortality worldwide. Several years after stroke, individuals may continue to experience cognitive impairments that significantly hinder their health-related quality of life (HRQOL) (1). Impairments in memory, attention, and executive functions are common but frequently underrecognised in post-stroke care (2). Even in the absence of motor or sensory sequelae, cognitive impairments can persist, continuing to diminish HRQOL over time (3, 4). Because these difficulties can be subtle and domain-specific, detection often requires neuropsychological evaluation. Impairments may not be immediately evident, yet they can still impact daily functioning (1). Cognitive problems are also reported as one of the most significant unmet needs in long-term recovery (5).

Despite the clinical relevance, research linking HRQOL with cognitive performance beyond the first year post-stroke remains scarce (1–3, 6). A recent systematic review and meta-analysis concluded that cognitive test performance is significantly linked to HRQOL after stroke, regardless of the time point for follow-up assessment (4). Small-to-moderate correlations were found between HRQOL and performance on cognitive tests across various cognitive domains, including processing speed, attention, visuospatial abilities, memory, and executive functions. However, language abilities did not show a significant relationship. The meta-analysis also highlighted weaknesses in the existing research. First, the assessment of cognition often relies on coarse screening tools like the Montreal Cognitive Assessment or the Mini-Mental State Examination, which lack sensitivity to subtle and domain-specific deficits (1). Second, the use of generic rather than stroke-specific HRQOL instruments limits the precision and relevance of findings (4).

The Stroke-Specific Quality of Life (SS-QOL) scale provides a broad, stroke-tailored assessment of HRQOL. Unlike generic HRQOL tools, it includes stroke-specific health components for physical functioning (PH), such as mobility and upper extremity function, as well as cognitive-social-mental functioning (CSM), which encompasses language, social roles, and mood (7, 8). Few prior studies have investigated the relationship between SS-QOL and cognitive test performance (4). Among these, only three included participants more than a year post-stroke (4, 6, 9, 10), two focused on individuals with subarachnoid haemorrhage (11, 12), and none included ischaemic stroke patients assessed with neuropsychological tests or cohorts from Scandinavia (10, 13).

To address this gap, the present study examined associations between neuropsychological test performance across a range of cognitive domains and the Norwegian version of the SS-QOL scale four years after stroke. We hypothesised that the CSM component would correlate more strongly with the cognitive tests than the PH component. This expectation was based on the conceptual overlap between CSM and cognitive domains. We expected significant associations for all domains except language. The strongest were hypothesised between CSM and processing speed and executive functions (3, 4, 14–18).

## Materials and methods

### Participant recruitment, study design and procedures

The present sample consisted of 65 participants from the Norwegian arm of the NorDenStroke multicentre study, a prospective observational cohort conducted in Northern Norway and Denmark. Details of the cohort have been published previously (7, 19). In brief, all patients with verified ischaemic or haemorrhagic stroke (ICD-10 codes I63 and I61) admitted to the stroke units at the University Hospital of North Norway (UNN-HF) between March 2014 and December 2015 were eligible. Exclusion criteria were stroke related to brain malignancy, subarachnoid haemorrhage, or brain trauma.

**Figure 1:**
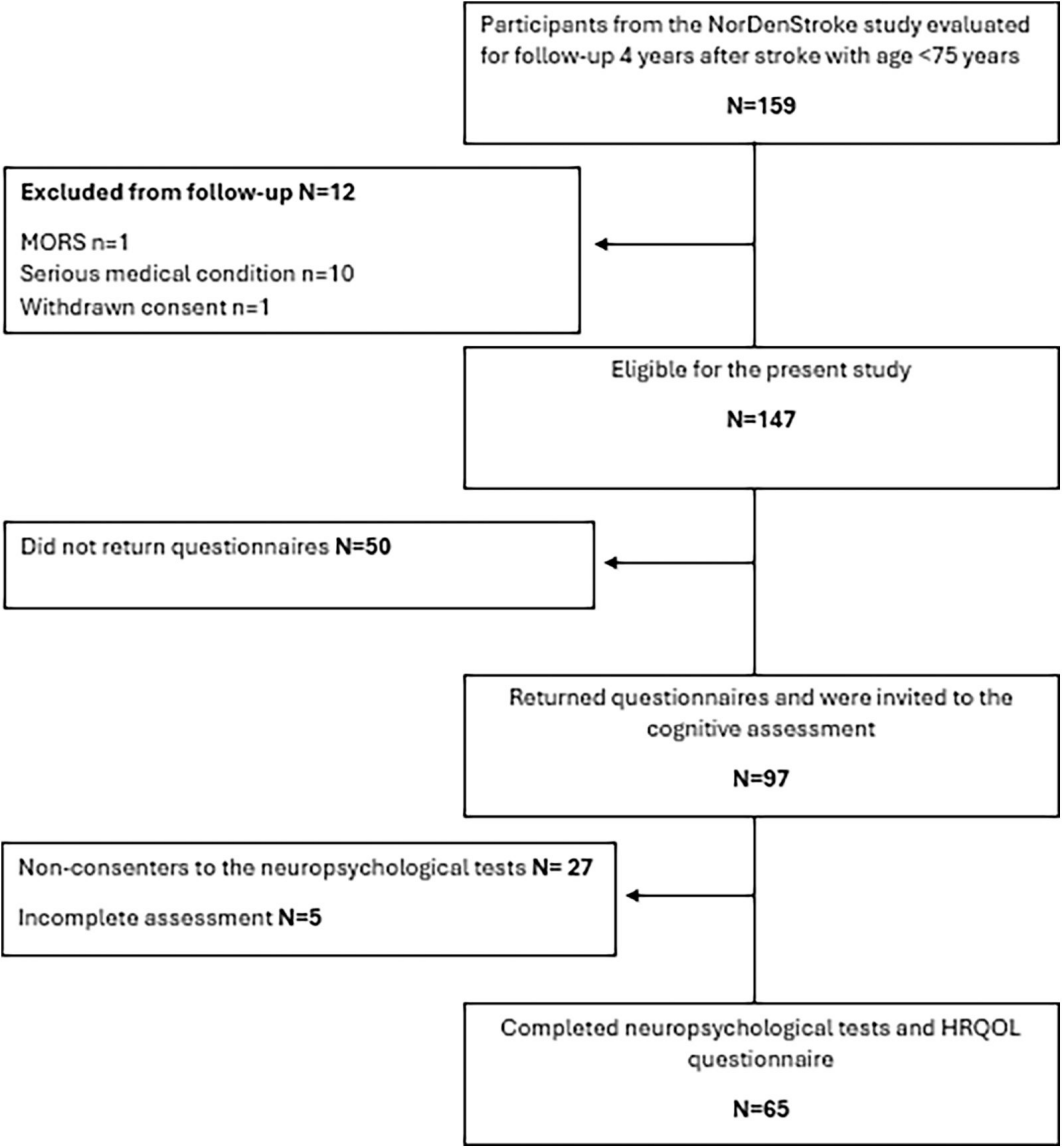
Flow chart. Inclusion, exclusion and loss of participants to the follow-up assessment.

At the 4-year follow-up, conducted between 2018 and 2019, all Norwegian participants younger than 75 years who had completed a previous 1-year follow-up (*N* = 159) were invited to participate. Participation consisted of completing questionnaires sent by mail, followed by an invitation to undergo an in-person neuropsychological assessment. Patients older than 75 years were not approached to reduce the impact of age-related, non-stroke cognitive decline. Of these, 97 returned mailed questionnaires. Following further exclusions due to aphasia, severe comorbidity, or non-consent, 65 participants completed an in-person neuropsychological assessment and were included in the present analyses. The participant flow is summarised in Figure 1.

Questionnaires were administered first by post, and neuropsychological testing was scheduled afterwards. We aimed to minimize the time interval between the two assessments, and for the 65 included participants, the median interval between questionnaire completion and neuropsychological testing was 28 days (IQR = 14–56 days). The scheduling of in-person testing was influenced by the considerable geographic distances in Northern Norway and, during winter, by challenging weather conditions. These logistical factors meant that appointments had to be arranged flexibly for each participant.

Neuropsychological evaluations were conducted in person by experienced clinical neuropsychologists or supervised psychology students with extensive training overseen by a specialist in neuropsychology. The sample in this paper is the same as that described in a previous publication investigating executive functions (18). Sample characteristics at 4-year follow-up and initial stroke characteristics of the participants are presented in Table 1. No participants experienced additional strokes in the four years leading up to the follow-up assessment.

**Table 1:** Sample characteristics at 4-year follow-up (*N* = 65).

Characteristic	*n* (%) or mean (SD)
Sex	Female: 17 (26) Male: 48 (74)
Age, years	64 (9)
Education	
≤10 years of schooling	20 (31)
High-school	25 (38)
Higher education	20 (31)
Living situation	With partner: 53 (82) Alone: 12 (18)
Work status	Working: 21 (32) Retired/sick leave/unemployed: 44 (68)
Modified rankin scale (mRS), 4-year follow-up	
No symptoms	11 (17)
No or mild disability	48 (74)
Moderate disability	5 (6)
Severe disability	1 (2)
Initial stroke characteristics	
Scandinavian stroke scale (SSS)	
Severe (15–29)	2 (3)
Moderate (30–44)	27 (42)
Mild (45–58)	36 (55)
Stroke type	Ischaemic: 61 (94) Haemorrhagic: 4 (6)

Sample characteristics at 4-year follow-up and initial stroke characteristics (*N* = 65). Values are presented as mean (SD) for continuous variables and *n* (%) for categorical variables.

The questionnaires and neuropsychological tests used in this study are described below

### Outcome measures

#### Stroke-specific quality of life (SS-QOL) scale

The SS-QOL scale is a comprehensive, stroke-specific tool designed to evaluate the impact of stroke on HRQOL. It includes 49 items spanning 12 subscales: mobility, energy, upper extremity function, work, mood, self-care, social roles, family roles, vision, language, thinking, and personality. Each subscale is assessed with three to six items on a 5-point Likert scale, where higher scores reflect better function. Subscale scores are averaged, and a total score can be derived across all domains. Previous studies have utilised principal component analysis to examine the scale's components, with Pedersen et al. (7) identifying two primary components that were labelled ‘physical health’ (PH) and ‘Cognitive-Social-Mental health’ (CSM). In the present study, component scores (CSM, PH) were computed as the mean of their constituent SS-QOL subscales following the loading structure reported by Pedersen et al. (7). No new factor analysis was performed. In addition, the total score is reported to facilitate comparison with other studies. The SS-QOL scale has demonstrated strong internal consistency and test-retest reliability in prior studies (7, 8, 19).

#### Modified rankin scale (mRS)

The modified Rankin Scale (mRS) was used to assess functional ability and independence in daily activities after stroke (20). The mRS is a widely applied global outcome measure ranging from 0 (no symptoms) to 6 (death) (20, 21). In this study, participants were provided with descriptive text for categories 1–5 to facilitate self-evaluation, an approach previously shown to yield valid results (22).

#### Neuropsychological assessment methods

We selected tests representing key cognitive domains commonly affected after stroke, including language, verbal memory, visuospatial abilities, fine-motor coordination, psychomotor speed, reaction time, attention, and executive functions (2, 16, 17). Eleven well-established neuropsychological tests, with age-adjusted, published normative data and official Norwegian translations, were used. From the Wechsler Adult Intelligence Scale 4th edition (WAIS-IV), we included Matrix Reasoning (visuospatial ability), Vocabulary (language), Coding (psychomotor speed) and Digit Span Backward (working memory) (23, 24). From the Conners’ Continuous Performance Test-3rd edition (CPT-III), the omission errors score was used to measure attention. In addition, the reaction time score was also selected from the CPT-III (25). From the California Verbal Learning Test, Second Edition (CVLT-II), the subtest Long-delay Free Recall was selected to represent verbal memory (26). The Grooved Pegboard Test (dominant hand, time to completion) was used to assess fine-motor coordination (27). From the Delis-Kaplan Executive Function System (D-KEFS), we used the Color Word Interference Test (Inhibition) and the Trail Making Test Number Letter Switching (Flexibility) and Letter Fluency was included as an additional language measure (28).

All tests were scored using published normative datasets based on large, age-stratified samples of healthy individuals (CVLT-II additionally adjusts for sex), as provided in the official manuals (24–28). These norms improve the precision of interpretation and allow stroke-related deficits to be distinguished from normal age-related changes. Because raw scores differ across tests (e.g., time to completion vs. number of correct responses), all results were converted to z-scores to provide a common metric (mean = 0, SD = 1), using the formula: z = (X−M)/SD, where X is the participant's raw score, M is the normative mean, and SD is the normative standard deviation. Where necessary, scores were reversed so that higher z-scores consistently indicate better performance.

### Statistics

Analyses were conducted in SPSS version 29. Descriptive statistics were computed for all variables, including SS-QOL scores and neuropsychological tests. Missing data occurred only for CVLT-II Long-delay Free Recall (*n* = 2) and D-KEFS Color Word Interference (*n* = 1); no imputation was applied. Normality of SS-QOL variables was assessed using Q-Q plots and skewness/kurtosis. The CSM component showed moderate deviation, and the PH component was highly skewed, reflecting a ceiling effect, with most participants reporting minimal physical disability. Consequently, associations were examined with Spearman's rho correlations. Higher scores indicated better functioning on all measures. To adjust for multiple testing, *p*-values were corrected with the Holm–Bonferroni procedure. Outliers (>3 SD from the mean) were detected for one participant on CPT-III and eight on the SS-QOL PH; exclusion did not alter results. Correlation coefficients were interpreted using standard thresholds (approximately.10 weak,.30 moderate,.50 strong).

## Results

As shown in Table 1, the 65 participants predominantly experienced mild to moderate strokes. Most were male, retired and living with a spouse. According to the mRS, the majority reported no symptoms or mild disability four years post-stroke.

Table 2 shows that participants performed below age-adjusted normative means across most cognitive domains. The lowest scores were seen in the domains of fine-motor coordination, reaction time and attention. Working memory was near the normative mean. Standard deviations indicated considerable variability, particularly in attention and cognitive flexibility, suggesting that while many participants performed within the normal range close to the normative mean, a subgroup demonstrated pronounced deficits.

**Table 2:** Descriptive statistics for cognitive test performance and SS-QOL scores (*N* = 65).

Domain	Test	Raw score mean (SD)	Age-adjusted z-score mean (SD)
Language	Letter fluency (D-KEFS)	31.78 (12.18)	−.43 (1.19)
	Vocabulary (WAIS-IV)	28 (9.79)	−.65 (.91)
Visuospatial ability	Matrix reasoning (WAIS-IV)	14.26 (5.71)	−.28 (1.13)
Psychomotor speed	Coding (WAIS-IV)	45.62 (15.28)	−.60 (.84)
Reaction time	Reaction time (CPT-III)	57.55 (11.27)^a^	−.76 (1.13)^b^
Attention	Omission errors (CPT-III)	57.08 (15.99)^a^	−.71 (1.69)^b^
Working memory	Digit span backward (WAIS-IV)	8 (2.28)	.07 (1.00)
Verbal memory	Long-delay Free recall (CVLT-II)	8.14 (3.14)	−.26 (.96)
Fine motor coordination	Grooved pegboard test, DH	106.72 (52.99)	−.89 (1.03)
Inhibition	Color word interference test (D-KEFS)	87.27 (36.03)	−.38 (1.29)
Flexibility	TMT number–letter switching (D-KEFS)	133.55 (63.69)	−.52 (1.34)
SS-QOL	Total score	Median = 4.53 (IQR 4.01–4.89)	—
	Physical health (PH)	Median = 4.95 (IQR 4.68–5.00)	—
	Cognitive–social–mental (CSM)	Median = 3.17 (IQR 2.41–3.52)	—

Raw scores are presented in their original test units. Raw scores were first adjusted for age using published normative datasets and subsequently transformed into z-scores (M = 0, SD = 1) to provide a common metric across tests.

^a^ CPT-III age-corrected T-scores because raw scores were not available.

^b^ The CPT-III T-scores were reversed and converted to z-scores so that higher values uniformly indicate better performance across cognitive measures. SS-QOL scores are raw scores, with higher values indicating better quality of life. SS-QOL, Stroke-specific quality of life; PH, physical health; CSM, cognitive-social-mental functioning; SD, standard deviation; IQR, interquartile range; D-KEFS, Delis-Kaplan executive function system; WAIS-IV, Wechsler adult intelligence scale—fourth edition; CPT-III, Conners’ continuous performance test—third edition; CVLT-II, California verbal learning test—second edition; DH, dominant hand, TMT: trail making test.

On the SS-QOL, PH scores were high with evidence of a ceiling effect, as most participants reported near-maximal scores. CSM scores were lower, reflecting persisting challenges.

Correlation analyses (Table 3) showed that better CSM scores were significantly associated with faster CPT-III reaction time, better verbal memory on the CVLT-II Long-delay Recall, and better fine-motor coordination on the Grooved Pegboard Test. These correlations were moderate to strong. PH scores showed a significant and moderately strong association with fine-motor coordination as measured with the Grooved Pegboard Test performance on the dominant hand. No significant associations were found for language, visuospatial ability, working memory, or executive functions (Inhibition, Flexibility) with either SS-QOL component.

**Table 3:** Spearman correlations (*ρ*, *p*) between cognitive test performance and SS-QOL scores (*N* = 65).

Domain	Test	PH–SS-QOL (*ρ*, *p*)	CSM–SS-QOL (*ρ*, *p*)
Language	Letter fluency (D-KEFS)	ρ = .11, *p* = .398	ρ = .31, *p* = .013
	Vocabulary (WAIS-IV)	ρ = −.11, *p* = .357	ρ = −.00, *p* = .987
Visuospatial ability	Matrix reasoning (WAIS-IV)	ρ = .16, *p* = .203	ρ = .25, *p* = .042
Psychomotor speed	Coding (WAIS-IV)	ρ = .22, *p* = .055	ρ = .32, *p* = .009
Reaction time	Reaction time (CPT-III)	ρ = .19, *p* = .137	**ρ** **=** **.47, *p*** **<** **.001**
Attention	Omission errors (CPT-III)	ρ = .15, *p* = .246	ρ = .26, *p* = .037
Working memory	Digit span backward (WAIS-IV)	ρ = .09, *p* = .471	ρ = .11, *p* = .291
Verbal memory	Long-delay free recall (CVLT-II)	ρ = .21, *p* = .095	**ρ** **=** **.41, *p*** **<** **.001**
Fine motor coordination	Grooved pegboard test, DH	**ρ** **=** **.49, *p*** **<** **.001**	**ρ** **=** **.39, *p*** **=** **.001**
Inhibition	Color word interference test (D-KEFS)	ρ = .15, *p* = .252	ρ = .27, *p* = .030
Flexibility	TMT number–letter switching (D-KEFS)	ρ = .15, *p* = .229	ρ = .23, *p* = .064

Correlations are based on age-adjusted z-scores for cognitive tests and raw scores for SS-QOL. *p*-values were corrected using the Holm–Bonferroni method. Significant correlations after correction are shown in bold with shaded cells. Higher scores indicate better functioning. SS-QOL, stroke-specific quality of life; PH, physical health; CSM, cognitive-social-mental; D-KEFS, Delis–Kaplan executive function system; WAIS-IV, Wechsler adult intelligence scale—fourth edition; CPT-III, Conners’ continuous performance test—third edition; CVLT-II, California verbal learning test—second edition; DH, dominant hand, TMT: trail making test.

## Discussion

Four years after stroke, participants reported preserved physical health (PH) but continued cognitive and psychosocial (CSM) challenges on the Stroke-Specific Quality of Life scale (SS-QOL). This CSM-PH discrepancy suggests that long-term Health-Related Quality of Life (HRQOL) cannot be accounted for by physical recovery alone and aligns with prior reports of persistent cognitive and psychosocial difficulties despite motor recovery (1, 3, 4). Neuropsychological performance varied. Most scored within the normal range, but a subgroup showed pronounced deficits.

The Grooved Pegboard Test was strongly correlated with both SS-QOL components and was the only cognitive test associated with PH. The Grooved Pegboard Test requires not only fine-motor skills but also visuo-motor integration, sequencing, and attentional control. These functions are essential for everyday activities such as cooking, hobbies, and digital communication. Reduced performance may therefore capture impairments across motor and cognitive systems, explaining its associations with both SS-QOL components (29).

Reaction time correlated with the CSM component. Simple reaction time tasks impose minimal motor demands, making them well-suited to detect slowed cognitive processing. Poorer performance has been linked to social and functional difficulties years after stroke (15) and is thought to reflect disruptions in attentional networks particularly vulnerable to stroke (14). Our findings further support the relevance of reaction time for HRQOL after stroke.

Our findings reinforce the importance of memory for long-term HRQOL. The verbal memory test was related to the CSM component, consistent with its importance for everyday functioning and social participation. Memory problems, such as forgetting conversations or appointments, may contribute to misunderstandings, reduced engagement, and eventual social isolation (1).

Executive functions (inhibition, working memory, flexibility) were not significantly associated with SS-QOL. This may reflect limited everyday demands in a predominantly retired sample, or insensitivity of the SS-QOL to subtle executive difficulties (16, 18). Other domains, including language, visuospatial ability, and attention, were also unrelated to SS-QOL.

Taken together, only three domains (reaction time, fine-motor coordination, and verbal memory) showed significant associations, suggesting that links between cognition and HRQOL may be circumscribed rather than broad. Nevertheless, these findings highlight the potential importance of addressing cognitive and psychosocial health as well as physical health in long-term stroke care. They also align with previous research showing that cognitive functioning remains important for HRQOL years after stroke (1–4, 16) and with research showing that cognitive and psychological problems are major unmet needs in long-term recovery (5). The observed associations between the Norwegian SS-QOL scale and cognitive performance provide some support for the instrument's validity, particularly the distinction between PH and CSM components (7, 8, 19).

### Strengths and limitations

This study has several strengths. To our knowledge, it is the first to examine associations between a more comprehensive neuropsychological test battery and the SS-QOL in an ischaemic stroke cohort. The long follow-up period of four years adds further novelty, as most studies have focused on the first year of recovery.

Nevertheless, limitations must be considered. The sample was small, community-dwelling, predominantly male, and characterised by mild-to-moderate strokes and high physical functioning. Older individuals (>75), individuals with aphasia or severe disability were excluded, further limiting representativeness. The type and extent of cognitive rehabilitation received were unknown, and findings may not generalise to other healthcare systems. While acute stroke care is generally of high quality in Northern Norway, systematic cognitive rehabilitation is not routinely offered for individuals without motor or sensory sequelae (30).

Despite these limitations, statistically significant associations emerged even after Holm–Bonferroni correction, with effect sizes comparable to or larger than those reported previously (4). Age-adjusted normative data enhanced the validity of neuropsychological scores, and outlier analyses confirmed that results were consistent, reflecting true clinical variation rather than error (see statistics). The strength of our findings likely reflects the use of standardised neuropsychological assessments and a stroke-specific HRQOL measure, both more sensitive than screening tools or generic HRQOL scales. As these findings are correlational, longitudinal studies are needed to clarify causality and the influence of potential confounders.

### Clinical implications and future directions

While motor recovery is often prioritised, long-term care should also address cognitive and psychosocial needs, which may persist despite minimal physical disability (3, 4). Routine cognitive screening in long-term follow-up may not be feasible for all, but clinicians should be alert to subtle cognitive difficulties and their potential impact on everyday functioning and well-being. Targeted interventions, including cognitive rehabilitation and psychosocial support, may help address these unmet needs. Future longitudinal studies with larger and more diverse samples are required to clarify causal pathways and identify which cognitive domains most strongly influence HRQOL at different recovery stages.

## Conclusion

Four years after stroke, this community-dwelling sample of mainly men younger than 75 years with mild-to-moderate events reported good physical recovery alongside persistent cognitive and psychosocial difficulties. Reaction time, verbal memory and fine motor coordination were the only domains associated with SS-QOL, suggesting that links between cognition and HRQOL are specific rather than general. Even in the absence of physical disability, focal cognitive difficulties may affect daily life, underscoring the need for targeted monitoring in long-term follow-up. The generalisability of the findings is limited by the small, relatively young and predominantly male sample, and by the exclusion of those with aphasia or more severe disability. Larger and more diverse studies are required to confirm these results.

## Data Availability

The data analyzed in this study is subject to the following licenses/restrictions: the dataset analyzed during the current study are not publicly available due to ethical restrictions and personal data protection. Data are available from the Data Protection Officer (contact personvernombudet@unn.no) for researchers who meet the criteria for access to confidential data. Requests to access these datasets should be directed to personvernombudet@unn.no.
